# Supplementary material for: Mutations in PCYT2 disrupt etherlipid biosynthesis and cause a complex hereditary spastic paraplegia
Source: Brain. 2019 Oct 22;142(11):3382–97. doi: 10.1093/brain/awz291 (PMC6821184; doi:10.1093/brain/awz291)
Supplement: awz291_Supplementary_Data [file awz291_supplementary_data.zip › awz291-Suppl_data/Supplementary_Data4.zip!/Supplementary information 3 - fibroblast lipidomics.zip!/Fibroblast lipidomics/Patient1 vs Patient2/Statistical plots and data/Volcano plot.pdf]

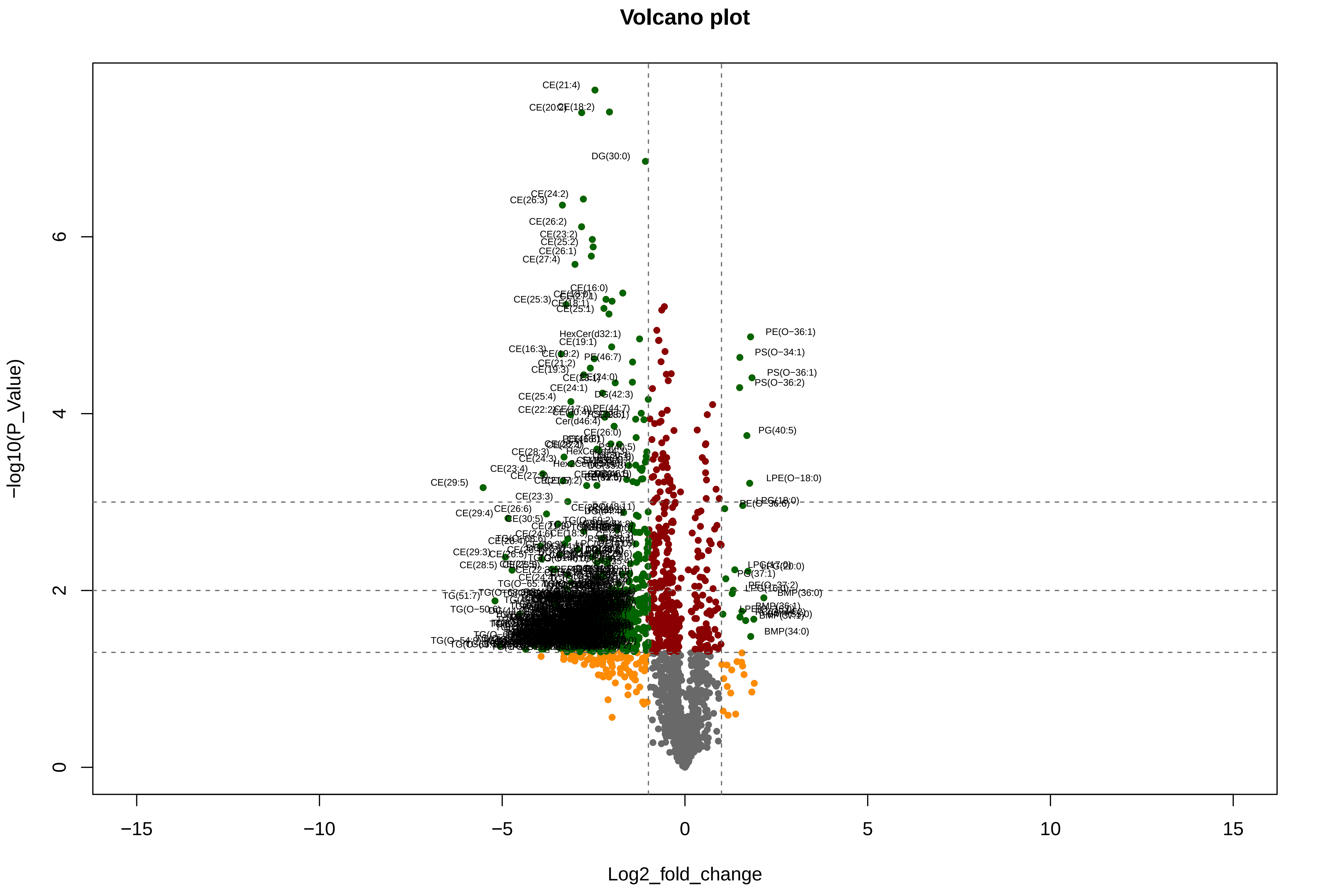

Volcano plot
−log10(P_Value)
Log2_fold_change
0
2
4
6
−15
−10
−5
0
5
10
15
CE(21:4)
CE(18:2)
DG(30:0)
CE(24:2)
CE(26:3)
CE(26:2)
CE(23:2)
CE(25:2)
CE(26:1)
CE(27:4)
CE(16:0)
CE(25:3)
CE(25:1)
HexCer(d32:1)
CE(19:1)
CE(16:3)
PE(46:7)
CE(21:2)
CE(19:3)
CE(24:1)
CE(25:4)
DG(42:3)
Cer(d46:4)
CE(26:0)
CE(28:3)
CE(24:3)
CE(23:4)
CE(29:5)
CE(23:3)
CE(26:6)
CE(29:4)
CE(30:5)
CE(29:3)
CE(28:5)
TG(51:7)
PE(O−36:1)
PS(O−34:1)
PS(O−36:1)
PS(O−36:2)
PG(40:5)
LPE(O−18:0)
PC(37:1)
BMP(36:0)
BMP(34:0)
